# Host Range Restriction and Pathogenicity in the Context of Influenza Pandemic

**DOI:** 10.3201/eid1206.051336

**Published:** 2006-06

**Authors:** Gabriele Neumann, Yoshihiro Kawaoka

**Affiliations:** * University of Wisconsin-Madison, Madison, USA;; † University of Tokyo, Tokyo, Japan;; ‡ Japan Science and Technology Agency, Saitama, Japan

**Keywords:** Influenza A virus, pathogenesis, host range, HA, NA, PB2, perspective

## Abstract

Influenza A viruses cause pandemics at random intervals. Pandemics are caused by viruses that contain a hemagglutinin (HA) surface glycoprotein to which human populations are immunologically naive. Such an HA can be introduced into the human population through reassortment between human and avian virus strains or through the direct transfer of an avian influenza virus to humans. The factors that determine the interspecies transmission and pathogenicity of influenza viruses are still poorly understood; however, the HA protein plays an important role in overcoming the interspecies barrier and in virulence in avian influenza viruses. Recently, the RNA polymerase (PB2) protein has also been recognized as a critical factor in host range restriction, while the nonstructural (NS1) protein affects the initial host immune responses. We summarize current knowledge of viral factors that determine host range restriction and pathogenicity of influenza A viruses.

## Abstract 2

Certain viral factors determine the host range restriction and pathogenicity of influenza A viruses.

Of the 3 types of influenza viruses (A, B, and C), only influenza A viruses are established in animals other than humans. Influenza pandemics are caused by viruses that have a hemagglutinin (HA) to which most humans have no immune memory. The strains of the 1957 Asian and 1968 Hong Kong pandemics had HAs derived from an avian virus. Although little information exists about avian influenza viruses at the time of the Spanish influenza pandemic, the HA of the virus responsible for that outbreak is also thought to be of avian origin. Since avian influenza viruses do not replicate efficiently in humans and nonhuman primates, they must overcome host range restriction for the avian virus HA to be introduced into human populations. The molecular basis for host range restriction is not well understood; however, HA plays a key role in the restriction of interspecies transmission.

The Spanish influenza was among the most devastating infectious diseases in history. At least 20 million people died worldwide. Antimicrobial agents were not available in 1918; however, existing evidence suggests that this high death toll was due to the extreme virulence of the virus. Although all 8 RNA segments of the Spanish influenza virus have been sequenced, these sequences offer no explanation for the high virulence. The Spanish influenza exemplifies how the magnitude of a pandemic can be determined by the pathogenicity of the virus.

In this review, we focus on 2 properties of influenza A viruses as they relate to pandemics, host range restriction and pathogenicity. Viral factors that affect these properties are examined.

## Viral Proteins Responsible for Host Range Restriction

### Viral Glycoproteins

The HA protein mediates virus binding to sialic acid (SA)–containing host cell surface molecules and promotes the release of viral ribonucleoprotein complexes through membrane fusion. By contrast, the sialidase activity of the neuraminidase (NA) protein removes SA to liberate newly synthesized viruses from infected cells. Thus, efficient virus replication requires the balanced actions of HA receptor-binding specificity and NA sialidase activity.

### HA Receptor Specificity

Influenza virus infectivity is influenced by 2 entities: SA species (N-acetylneuraminic acid [NeuAc] and N-glycolylneuramic acid [NeuGc]) and the type of linkage to galactose (sialyloligosaccharides terminated by SA linked to galactose by an α2,6 linkage [Acα2,6Gal] or an α2,3 linkage [Acα2,3Gal]) on the host cell surface. Human influenza viruses preferentially recognize sialyloligosacchrides containing SAα2,6Gal (*1**,**2*), matched by mainly NeuAcα2,6Gal linkages on the epithelial cells of the human trachea (*3*). By contrast, avian viruses preferentially recognize SAα2,3Gal sialic acids (*1**,**2*), in accordance with the predominance of sialyoligosaccharides with SAα2,3Gal linkages on the epithelial cells of duck intestine. The epithelial cells of pig trachea contain both types of SAs and both types of linkages (*4*), which likely explains the high susceptibility of these animals to both human and avian influenza viruses (*5*). Pigs may therefore serve as a "mixing vessel" for reassortment between these 2 viruses and the source of pandemic strains, although no evidence exists that the 1957 or 1968 pandemic viruses originated in pigs.

Despite these differences in receptor specificity, avian viruses can infect humans and have caused lethal infections (*6**–**8*). This fact may be explained by the recent finding that in differentiated cultures of human tracheobronchial epithelium, α2,3-linked SAs were found on ciliated cells, whereas α2,6-linked SAs were present on nonciliated cells (*9*). The prevalence of cells that possess α2,6 and/or α2,3-linked SAs in the lower respiratory tract remains unknown; however, ciliated cells support avian virus infection. Despite the presence of α2,6-linked SA-bearing cells in differentiated human tracheobronchial epithelium, viruses with avian-type receptor specificity can infect humans, since the index 1997 H5N1 virus isolated from a human preferentially recognized an avian receptor (*10*). Nevertheless, for efficient human-to-human transmission, HA derived from an avian virus must preferentially recognize the human receptor. This notion is supported by the finding that the earliest isolates in the 1918 (*11**,**12*), 1957, and 1968 pandemics preferentially recognized NeuAcα2,6Gal–containing sialyloligosaccharides (*13*), even though their HAs were derived from avian viruses. Conversion of receptor specificity from SAα2,3Gal to SAα2,6Gal may therefore be critical for generating pandemic influenza viruses.

Sequence comparison, receptor specificity assays, and crystallographic analysis have identified amino acid residues that determine receptor specificity: Gln-226 (found in avian viruses) determines specificity for SAα2,3Gal, whereas Leu-226 correlates with SAα2,6Gal specificity in human H2 and H3, but not H1, viruses (*2**,**13*). In all human viruses (with the few exceptions of early isolates from the Asian influenza outbreak [13]), Leu-226 is associated with Ser-228, while Gln-226 is associated with Gly-228 in avian viruses. For H1 viruses, Asp-190 (found in human and swine virus isolates) or Glu-190 (found in avian virus isolates) determines preferential binding to α2,6 or α2,3 linkages, respectively (*11**–**14*).

Land-based poultry are thought to play a critical role in the emergence of pandemic influenza viruses. Compared to H5N1 viruses isolated from aquatic birds, those isolated from chickens have significantly lower affinity for NeuAcα2,3Gal (*10*), similar to human virus isolates; however, H5N1 chicken isolates have not acquired preferential specificity for NeuAcα2,6Gal. H5N1 chicken isolates with reduced avian receptor specificity share 2 characteristic features of human viruses, namely, an additional glycosylation site in the globular head region of HA and a deletion in the NA stalk (see below). Similarly, the receptor specificity of H9N2 viruses isolated from land-based poultry, but not of those isolated from aquatic birds, is similar to that of human isolates (*15*). Hence, land-based poultry may serve as an intermediate host that facilitates the conversion of avian to human-type receptors. Avian viruses in land-based poultry may, therefore, pose a greater threat to humans than previously thought.

### NA Properties

Since efficient release of virus from infected cells requires the removal of SA by NA, the receptor-binding and receptor-destroying properties of HA and NA, respectively, must be balanced. When an avian virus with an N2 NA was introduced into the human population, its SAα2,6 cleavage activity increased (*16**,**17*), which suggests it had adapted to the SAα2,6 receptor specificity of human HAs.

The NA stalk, which separates the head region with the enzymatic center from the transmembrane and cytoplasmic domains, varies in sequence and length, depending on the virus (*18*). Typically, shortened stalks result in less efficient virus release since the active site in the head region cannot efficiently access its substrate (*19**,**20*). However, naturally occurring avian viruses with shortened stalks are virulent in poultry, and the 1997 H5N1 viruses isolated from patients in Hong Kong (which are believed to have been transmitted to humans from poultry) are characterized by a deletion in the NA stalk (*10*). Moreover, most recent highly pathogenic H5N1 viruses isolated from terrestrial poultry possess short NA stalks (*21*).

In avian species, the intestinal tract is the primary site of replication, whereas in humans, influenza virus replication is typically restricted to the respiratory tract. The NA activity of avian H1N1 viruses is more resistant to the low pH environment in the upper digestive tract than is its human or swine-derived counterpart (*22*). In line with this finding, highly pathogenic H5N1 viruses can replicate in the human intestine, causing gastrointestinal symptoms (*23*), and are shed in large amounts in stool.

### Internal Proteins

Classical coinfection experiments, or reverse genetics experiments that tested multiple gene combinations of 2 parental viruses, suggest that the genes encoding the "internal proteins"—namely, RNA polymerase (PB2, PB1, PA), nucleoprotein (NP), matrix protein (M1, M2), and nonstructural protein (NS1, NS2/NEP)—also contribute to host range. The contribution of individual proteins to host range restriction, however, likely varies, depending on the test system and the virus strains under investigation.

### PB2

PB2 is a component of the viral polymerase complex and, as such, is essential for viral replication. The 1997 H5N1 human virus isolates in Hong Kong have been divided into 2 groups on the basis of their pathogenicity in mice; this classification also generally corresponds to disease severity in humans (*24**,**25*). Reverse genetics studies have shown that Lys at position 627 of PB2 (found in all human isolates) determines high pathogenicity in mice, while Glu at this position (found in all avian isolates) determines low pathogenicity (*26*). However, the nature of the amino acid at position 627 of PB2 does not affect the cell tropism of the virus but rather its replicative ability in mice and probably in humans.

Several other findings underline the importance of residue 627 of PB2: 1) an H7N7 virus isolated from a patient with fatal pneumonia in the Netherlands in 2003 contained Lys at this position, in contrast to viruses isolated from nonfatal cases and from chickens (*27*); 2) some of the H5N1 viruses isolated from patients in Vietnam are characterized by Lys-627 in PB2 (*28*); 3) a single reassortant virus bearing an avian virus PB2 gene against a human virus background replicated efficiently in avian but not human cells, a feature that could be traced to the nature of the amino acid at position 627 of PB2 (*29*); and 4) ribonucleoprotein complexes reconstituted from human or avian polymerase and NP proteins identified residue 627 of PB2 as the major determinant of replication efficiency in mammalian cells (*30*). Collectively, these findings suggest that a Glu-to-Lys mutation at position 627 of the PB2 protein allows avian viruses to efficiently grow in humans and implicates Lys at this position as an important host range determinant.

### Other Components of the Replication Complex

In addition to PB2, the remaining 2 polymerase proteins (PB1 and PA) and the nucleoprotein NP may also contribute to host range. In a minireplicon system, replication in mammalian cells was more efficient with avian than with human virus PB1 proteins (*30*), which suggests that avian PB1 may have greater activity that could provide a replicative advantage in mammalian systems. This scenario is especially appealing in light of the finding that both the 1957 and 1968 pandemic viruses possessed avian PB1 genes, in addition to avian HA, NA, or both genes (*31**,**32*). In one study, however, an avian PB1 gene severely restricted replication of a human virus in mammalian cells and squirrel monkeys (*33*). These findings seem to contradict a role of avian PB1 in replication in mammalian cells. PA and NP proteins have also been implicated in host range restriction; for example, an avian virus NP segment against a background of a human virus resulted in attenuation in squirrel monkeys (*33*). However, because of the limited data available, whether these findings indicate a contribution of these gene products to host range restriction or simply reflect incompatibility among the viral gene segments is unclear.

### M Segment

Segment 7 of influenza A viruses encodes the M1 matrix and the M2 ion channel proteins. In coinfection experiments that selected for reassortants containing a human virus M gene and an avian virus HA gene, the M segment of an early human virus (A/PR/8/34, H1N1) cooperated efficiently with avian virus HAs, whereas M segments derived from more recent isolates have gradually lost this ability (*34*). This finding may suggest that currently circulating human viruses are less likely to reassort with avian viruses than their predecessors. If this is the case, the risk for a global pandemic caused by reassortants possessing avian HA, NA, or both segments against a human virus background would be reduced.

## Molecular Basis of Pathogenicity

### HA Cleavability

The HA protein is synthesized as a precursor protein that is cleaved into 2 subunits (HA1 and HA2) by host cell proteases. HA cleavage is a prerequisite for fusion of the viral and endosomal membranes and, therefore, for viral infectivity (*35*). Low pathogenic avian influenza viruses possess a single Arg residue at the cleavage site, recognized by extracellular, trypsinlike proteases. These proteases are thought to be secreted only by cells of the respiratory and intestinal tract and consequently limit infections to these organs. By contrast, highly pathogenic avian viruses possess multiple basic amino acids at the cleavage that are recognized by ubiquitous, intracellular, subtilisin-like proteases that thus trigger systemic infection. In addition, HA cleavability is affected by the absence or presence of a carbohydrate side chain near the cleavage site that may interfere with the accessibility of host proteases to the cleavage site (*36*). The acquisition of a highly cleavable HA converted an avirulent strain to virulence in Pennsylvania in 1983 (H5N2), Mexico in 1994 (H5N2), Italy in 1997 (H7N1), Chile in 2002 (H7N3), and Canada in 2004 (H7N3) (Table). HA cleavability is, therefore, considered the major determinant of tissue tropism of avian influenza viruses (*41*). This correlation seems to extend to humans, since all avian viruses that have killed humans possess a highly cleavable HA (*6**,**7**,**27*), and an H5N1 mutant virus whose HA cleavage site had been changed to an avirulent type was attenuated in mice (*26*).

**Table:** Comparison of the hemagglutinin (HA) cleavage sites of highly pathogenic avian influenza viruses and their nonpathogenic predecessors

Isolate	Type	Amino acid sequence*	Reference
A/chicken/Pennsylvania/1/83 (H5N2)	Avirulent	P Q - - - - - - - - - - - K K K R/ G L F	(*36*)
A/chicken/Pennsylvania/1370/83 (H5N2)	Virulent	P Q - - - - - - - - - - - K K K R/ G L F†	(*36*)
A/chicken/Mexico/31381-7/94 (H5N2)	Avirulent	P Q - - - - - - - - - - - R E T R/ G L F	(*37*)
A/chicken/Queretaro/14588-19/95 (H5N2)	Virulent	P Q - - - - - - - - - R K R K T R/ G L F	(*37*)
A/turkey/Italy/99 (H7N1) consensus	Avirulent	P E I P K G - - - - - - - - - - R/ G L F	(*38*)
A/turkey/Italy/99 (H7N1) consensus	Virulent	P E I P K G - - - - - - S R V R R/ G L F	(*38*)
A/chicken/Chile/176822/02 (H7N3)	Avirulent	P E K P K - - - - - - - - - - T R/ G L F	(*39*)
A/chicken/Chile/4957/02 (H7N3)	Virulent	P E K P K T C S P L S R C R K T R/ G L F	(*39*)
A/chicken/Chile/4322/02 (H7N3)	Virulent	P E K P K T C S P L S R C R E T R/ G L F	(*39*)
Isolate CN6/04	Avirulent	P E N P K - - - - - - - - - - T R/ G L F	(*40*)
A/chicken/BC/CN12/04(H7N3)	Virulent	P E N P K - - --Q A Y Q K R M T R/ G L F	(*40*)
A/chicken/BC/NS1337-1/04 (H7N3)	Virulent	P E N P K - - - Q A Y K K R M T R/ G L F	(*40*)
A/chicken/BC/NS-1319-2/04(H7N3)	Virulent	P E N P K - - - Q A Y H K R M T R/ G L F	(*40*)
A/chicken/BC/CN7-3/04 (H7N3)	Virulent	P E N P K - - - Q A Y R K R M T R/ G L F	(*40*)
A/chicken/BC/NS-1390-2/04(H7N3)	Virulent	P E N P K - - - Q A H Q K R M T R/ G L F	(*40*)
A/chicken/BC/NS-2035-12/04(H7N3)	Virulent	P E N P K - - - Q A C Q K R M T R/ G L F	(*40*)

*HA cleavage sites are indicated by /. For sequence variants, the amino acids that differ from most sequences found are underlined. †HA cleavability was enhanced by a single amino acid substitution that abrogated glycosylation near the HA cleavage site.

### Role of NS1 in Antagonizing Cellular Immune Responses

Pathogenesis depends partly on the ability of a virus to evade or suppress the host immune response. The NS1 protein, encoded by segment 8, plays a central role in this process by counteracting the cellular interferon (IFN) response in a 2-pronged approach: 1) by binding to double-stranded RNA, thereby suppressing the activation of double-stranded RNA-activated protein kinase, a known stimulator of type I IFN, and 2) by preventing the activation of transcription factors such as ATF-2/c-Jun, NFκB, and IRF-3/5/7, all of which stimulate IFN production (*42**,**43*). The NS gene of the 1918 Spanish flu blocked the expression of IFN-regulated genes in human cells more efficiently than did the NS gene of the A/PR/8/34 (H1N1) virus (*44*), which suggests that the NS genes of highly pathogenic viruses may be more proficient in counteracting the host immune response than those of less pathogenic viruses.

Viruses containing the NS gene of the 1997 H5N1 virus are potent inducers of proinflammatory cytokine genes, particularly tumor necrosis factor-α (TNF-α) and IFN-β in human primary monocyte-derived macrophages (*45*). Similarly, 2003 human H5N1 isolates induce high levels of proinflammatory cytokines in primary human macrophages (*46*). These in vitro findings are substantiated by reports of unusually high serum concentrations of chemokines in patients infected with H5N1 influenza viruses. High levels of macrophage-derived chemokines and cytokines were also induced by a recombinant virus containing a gene segment of the 1918 Spanish flu; in this case, however, the HA segment stimulated the increased levels of chemokines and cytokines (*14**,**47*). This upregulation of cytokine function at later phases of infection may account for the unusual clinical signs and symptoms and the degree of disease severity associated with human infections of highly pathogenic influenza viruses.

Highly pathogenic H5N1 viruses not only trigger the overproduction of proinflammatory cytokines but also are resistant to the antiviral effects of IFN and TNF-α. Pretreatment of porcine lung epithelial cells with IFN-α, IFN-γ, or TNF-α has no effect on the replication of a recombinant human H1N1 virus possessing the NS gene of the 1997 H5N1 virus but abolishes replication of the parental human H1N1 virus (*48**,**49*). Resistance to the antiviral effects of IFN and TNF-α is associated with glutamic acid at position 92 of the NS1 protein, as demonstrated by reverse genetics studies. These in vitro data extend to in vivo findings, since pigs infected with a virus containing Glu-92 in NS1 experience higher virus titers and body temperatures than those infected with a control virus (*48**,**49*). Collectively, these findings indicate that NS1 induces a cytokine imbalance that likely contributes to the extreme pathogenicity of avian influenza viruses in humans.

## Conclusions

One might speculate that the next pandemic may be caused by highly pathogenic H5N1 viruses that acquire the ability to be efficiently transmitted among humans, or by H9N2 viruses, which are as prevalent as H5N1 viruses in Asia and in some cases already recognize human receptors. Further investigation of the molecular basis of host range restriction is therefore important. In addition, a better understanding of the mechanisms and consequences of chemokine/cytokine imbalance caused by highly pathogenic avian viruses is essential, as is a greater appreciation for the contributions of other viral properties, such as replicative ability, to pathogenesis.
